# Ultrasonography of a Helical Left Common Carotid Artery

**DOI:** 10.5811/cpcem.2020.2.46272

**Published:** 2020-04-23

**Authors:** Bethany J. Busack, Vy Tran, Christopher D. Busack, Christine J. Butts

**Affiliations:** * Louisiana State University Health Sciences Center, Department of Emergency Medicine, New Orleans, Louisiana; † Ochsner Clinic Foundation, Department of Anesthesiology, New Orleans, Louisiana; ‡ Tulane University School of Medicine, Department of Anesthesiology, New Orleans, Louisiana

**Keywords:** Ultrasound, central venous access, internal jugular vein, anatomy

## Abstract

**Case Presentation:**

An 83-year-old woman was admitted to the intensive care unit for septic shock at which point an internal jugular central venous line was placed. The patient’s common carotid artery was visualized in an atypical location, lateral to the internal jugular vein. Further inspection revealed the common carotid artery travelling in a rotational trajectory around the internal jugular vein.

**Discussion:**

For at least two decades, point-of-care ultrasound has become the standard of care for placing central venous lines. This surprising anatomical orientation is rare and cautions physicians to fully explore a patient’s anatomy prior to placing central lines.

## CASE PRESENTATION

An 83-year-old woman was admitted to the intensive care unit for septic shock. During central venous catheter placement, ultrasonography was used to guide insertion. When the probe was placed on the left side of the neck in a neutral position, the internal jugular vein (IJV) was noted to be medial to the common carotid artery (CCA) (Image, Panel A). Upon scanning caudad approximately eight centimeters, the CCA coursed medially in a rotational trajectory nearly 180 degrees (Video) ending up in its typical orientation, medial to the IJV (Image, Panel B and C).

## DISCUSSION

The variability in CCA and IJV orientation has been studied with multiple imaging modalities. In a series of 188 patients undergoing ultrasonography, only one patient demonstrated an IJV in the medial position.^1^ Rotation of the CCA and IJV has not been described to our knowledge. However, one case report describes a duplicated IJV, with the medial branch crossing the CCA.^2^ A magnetic resonance angiography study found increasing age to be positively correlated with vessel tortuosity.^3^ Although it did not comment on position of the CCA in reference to the IJV, this study suggests that the anatomy of the carotid artery in an elderly patient, such as the patient in this case, may not follow the typical configuration. Delineating the full extent of the patient’s particular anatomy prior to needle insertion, perhaps particularly in older patients, helps to avoid inadvertent arterial puncture and increase successful venipuncture. Additional parameters, such as vessel compressibility and wall thickness, should be used in conjunction with the traditional anatomic orientation to properly identify the vein from the artery prior to cannulation.

**CPC-EM Capsule**

What do we already know about this clinical entity?

The anatomic location of the common carotid artery is typically medial to the internal jugular vein, however may lie posteriorly, anteriorly, or rarely laterally.

What is the major impact of the image(s)?

The image reiterates the importance of using multiple modalities, rather than location alone, to ensure proper identification of the internal jugular vein prior to cannulation.

How might this improve emergency medicine practice?

The image encourages physicians to fully scan the neck prior to venous cannulation in order to identify the correct vessel and delineate atypical venous anatomy.

## Supplementary Information

Video With the provider at the head of the bed with the ultrasound probe on the patients left neck, we initially see the common cartoid lateral to the internal jugular vein. Sliding the probe caudad, the artery dives in a spiral trajectory posterior to the vein, ultimately reaching its typical orientation medial to the vein just above the clavicle.

## Figures and Tables

**Image:**
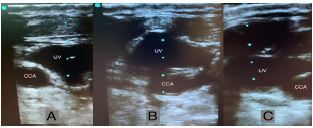
(A) Ultrasonography of patient’s left neck in a neutral position showing the common carotid artery (CCA) lateral to the internal jugular vein (IJV), (B) coursing posteriorly as it travels caudad in a rotational trajectory, and (C) ultimately reaching its typical orientation medial to the IJV.
